# Comparison of the Intrauterine Enabler Device Technique and Conventional Method for Inserting Copper 380 mm² Intrauterine Device: A Pilot Randomized Controlled Trial

**DOI:** 10.7759/cureus.70687

**Published:** 2024-10-02

**Authors:** Latika Chawla, Sharad Singh, Surekha Thakur, Mamta K Sah, Shashi Prateek, Jaya Chaturvedi

**Affiliations:** 1 Obstetrics and Gynecology, All India Institute of Medical Sciences, Rishikesh, Rishikesh, IND; 2 Obstetrics and Gynecology, Pregna International Ltd., Delhi, IND

**Keywords:** contraception, copper 380mm2 iud, intrauterine enabler device, iud insertion, no touch technique

## Abstract

Background

This pilot study compared the intrauterine enabler device technique with the conventional method for inserting the copper 380 mm² intrauterine device (Cu-IUD), focusing on the fundal placement achieved by both methods.

Methods

This randomized controlled trial compared the provider and client experiences as well as the technique for inserting the Cu-IUD using the intrauterine enabler device and the conventional no-touch technique. A total of 100 clients underwent Cu-IUD insertion: 50 participants (Group A) received the conventional no-touch technique, while 50 participants (Group B) were treated with the intrauterine enabler device.

Results

Pain experienced during sounding with the Esa (plastic sound provided with an inserter device) was significantly lower compared to the metallic sound (p < 0.0001). Providers in Group B reported that loading the arms of the Cu-IUD was easy 94% of the time, compared to only 26% in Group A (p < 0.0001). Setting the measurement of uterine depth on the inserter tube was achieved easily every time in Group B and 90% of the time in Group A (p = 0.054). The placement technique for the device was easy every time in Group B, while it was reported as easy 86% of the time in Group A (p = 0.012). Overhandling of the device was significantly easier with the intrauterine enabler (98%) compared to the conventional method (64%) (p < 0.0001). Additionally, the time taken for insertion was significantly shorter with the intrauterine enabler compared to the conventional method (p < 0.0001).

Conclusions

The insertion of the Cu-IUD using a novel intrauterine enabler device is as safe and effective as the conventional method. The overall experience of insertion is more comfortable for the provider and less painful for the client.

## Introduction

Intrauterine contraceptive devices (IUCDs) are the most commonly used method of contraception worldwide, offering long-acting reversible options. They are particularly preferred by women seeking to space pregnancies, those who are breastfeeding, individuals with medical conditions necessitating pregnancy postponement, and those who face challenges with other temporary contraceptive methods. IUCDs also do not interfere with sexual activity [1].

The copper 380 mm² intrauterine device (Cu-IUD) is a long-acting, reversible contraceptive method. According to data from the National Family Health Survey 5, approximately 2.1% of women in India aged 15-45 utilize IUDs for contraception [2]. The Cu T 380 A is a T-shaped device constructed with a polyethylene frame, providing 380 mm² of exposed copper surface area. This includes 314 mm² of copper wrapped around the vertical stem and 33 mm² on the transverse arms [1,3]. The IUD frame incorporates barium sulfate, rendering it radio-opaque. A trained healthcare provider inserts the IUD into the uterus through the vagina and cervix.

To minimize the risk of infection during the insertion of the Cu 380 mm² IUD, the WHO developed the “no-touch technique” [4]. This technique involves ensuring that the IUD does not come into contact with any surface, including the provider’s gloved hands, the speculum, the vagina, or the tabletop during insertion. The IUD is loaded into the inserter while remaining in its sterile packaging, avoiding direct contact. Care is taken to prevent the IUD from touching the vaginal walls or speculum blades during insertion. The sound and loaded Cu-IUD should be passed through the cervical canal only once, and the Cu-IUD should be placed within the uterine cavity following the instructions on the label [5,6].

The intrauterine enabler device is an innovative tool that features the Cu-IUD mounted on a disposable plastic frame [7]. It is designed to streamline the aseptic loading of the Cu-IUD into the inserter tube, aiming to reduce client waiting time and enhance the overall experience for both providers and users by making the process safer and faster. The device enables insertion of the Cu-IUD using a withdrawal method, which minimizes the risk of uterine perforation and ensures satisfactory fundal placement [7].

## Materials and methods

This pilot study compares the insertion technique of the Cu-IUD using the novel intrauterine enabler device with the conventional method, as well as the fundal placement of the Cu IUD achieved by both methods. The primary objective is to compare the insertion technique of the Cu-IUD using the novel intrauterine enabler device with the conventional method, while the secondary objectives include comparing client comfort and feedback in the two groups and assessing fundal placement achieved with both methods.

This randomized controlled trial was conducted in the Department of Obstetrics and Gynecology at the All India Institute of Medical Sciences (AIIMS), Rishikesh, India. Institutional ethical committee approval was obtained (approval numbers AIIMS/IEC/19/1284 and AIIMS/IEC/20/810). The trial was registered with the Clinical Trials Registry - India (CTRI/2020/06/025578). The study was conducted over a period of 16 months, from July 2020 to October 2021. Women attending the OPD seeking contraceptive advice were counseled using the CAFETERIA approach, where a variety of contraceptive options were offered to them [8].

The study included parous women aged 20-45 years who chose the Cu-IUD as their interval contraceptive method and consented to participate. We excluded women with uterine abnormalities, such as congenital anomalies, active pelvic inflammatory disease, endometrial lesions, cervical or endometrial cancer, suspected pregnancy, or intrauterine adhesions. Additionally, women undergoing Cu T insertion immediately after delivery, postpartum, or following an abortion were excluded. Informed written consent was obtained from all eligible participants after explaining the study’s nature.

Participants were randomized into two groups using simple randomization via a lottery method (flipping a coin). Participants undergoing Cu-IUD insertion by the conventional no-touch technique were assigned to Group A, while those undergoing Cu IUD insertion with the Intrauterine Enabler device (Etherena T Cu 380 A, Pregna International Ltd., Delhi, India) were assigned to Group B. Given that this was a pilot project, a sample size of 100 participants was established, with 50 participants allocated to each group. Doctors with a minimum of one year of experience in Cu-IUD insertion performed the procedures. A training session was organized for the doctors, during which the insertion technique using the intrauterine enabler device was demonstrated through a video presentation, followed by hands-on practice using simulation. Informed written consent was obtained from all participants. The insertion of the Cu-IUD was performed in the OPD with all sterile precautions, and a complete pelvic examination was conducted prior to the insertion.

**Figure 1:**
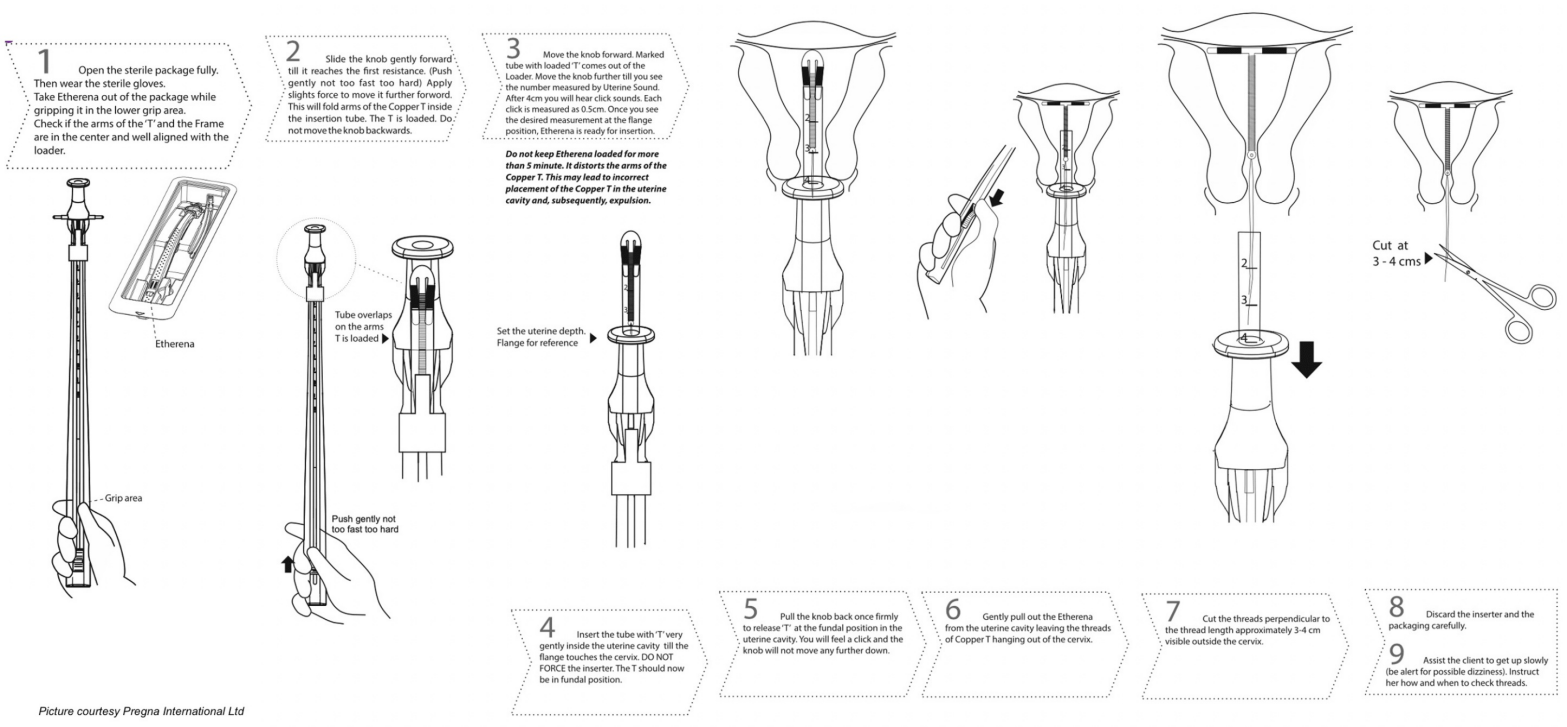
Steps for the insertion of the Copper T IUD using the intrauterine enabler device IUD, intrauterine device Source: Pregna International Ltd. (reprinted with permission)

The technique for inserting the Cu-IUD using the intrauterine enabler device (Etherena T Cu 380 A) is demonstrated stepwise in Figure 1.

**Figure 2:**
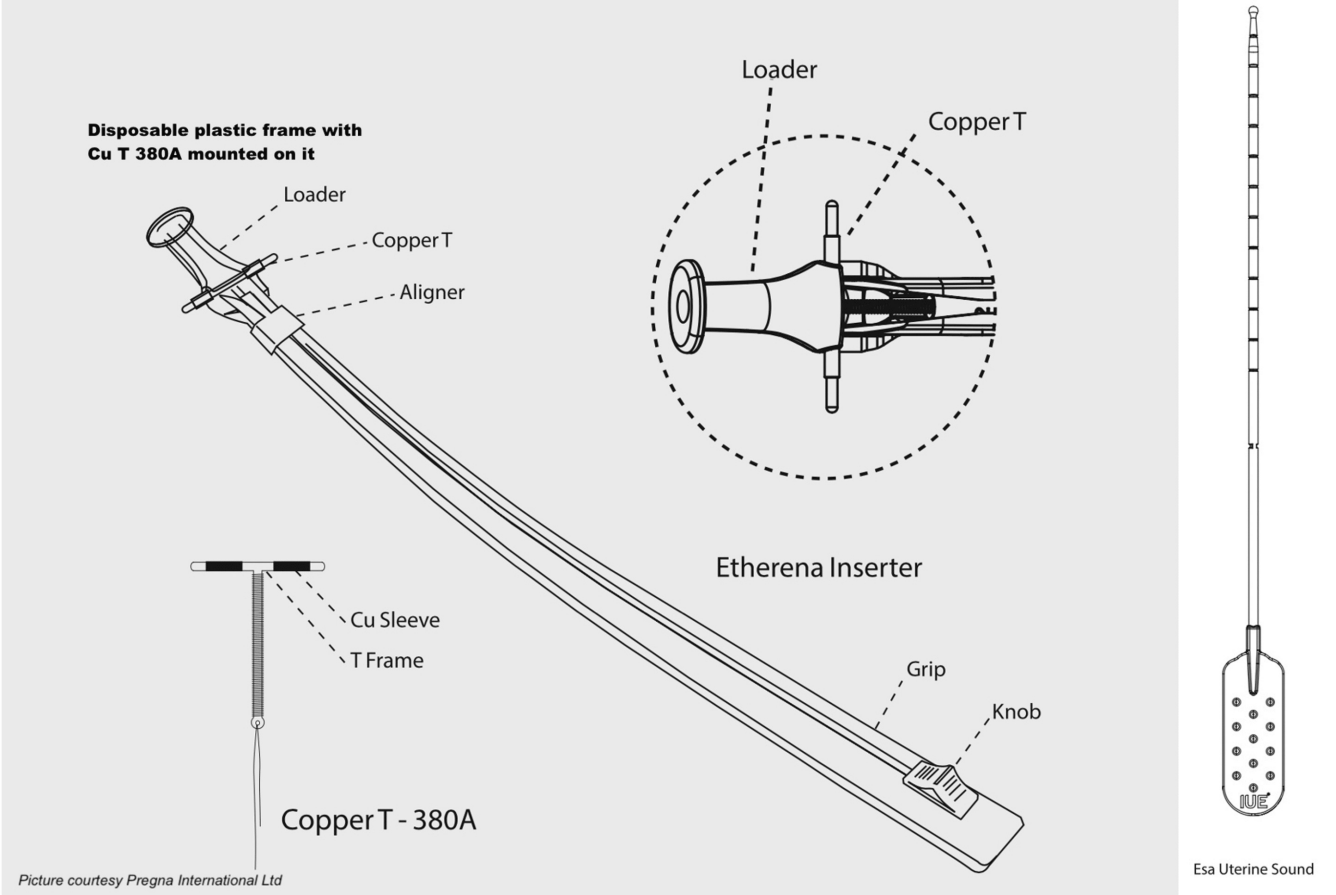
Intrauterine enabler device (Etherena T Cu 380 A) Source: Pregna International Ltd. (reprinted with permission)

The enabler device features a disposable plastic frame with the Cu-IUD mounted on it, along with a disposable plastic sound (Esa) (Figure 2).

Conventional method for the insertion of Cu T

In the conventional method, the Cu-IUD was loaded and inserted using the “no-touch technique” recommended by the WHO [3]. This technique is well-practiced by doctors at our institute, as it is included in the postgraduate training curriculum and routinely utilized in clinical practice. The uterocervical length was measured with a metallic sound, and fundal placement of the Cu-IUD was confirmed via transvaginal ultrasound immediately after insertion and again at one month of follow-up. Proper placement was defined as being within 2 mm of the endometrial verge, measured from the superior end of the Cu-IUD to the endometrial verge.

**Figure 3:**
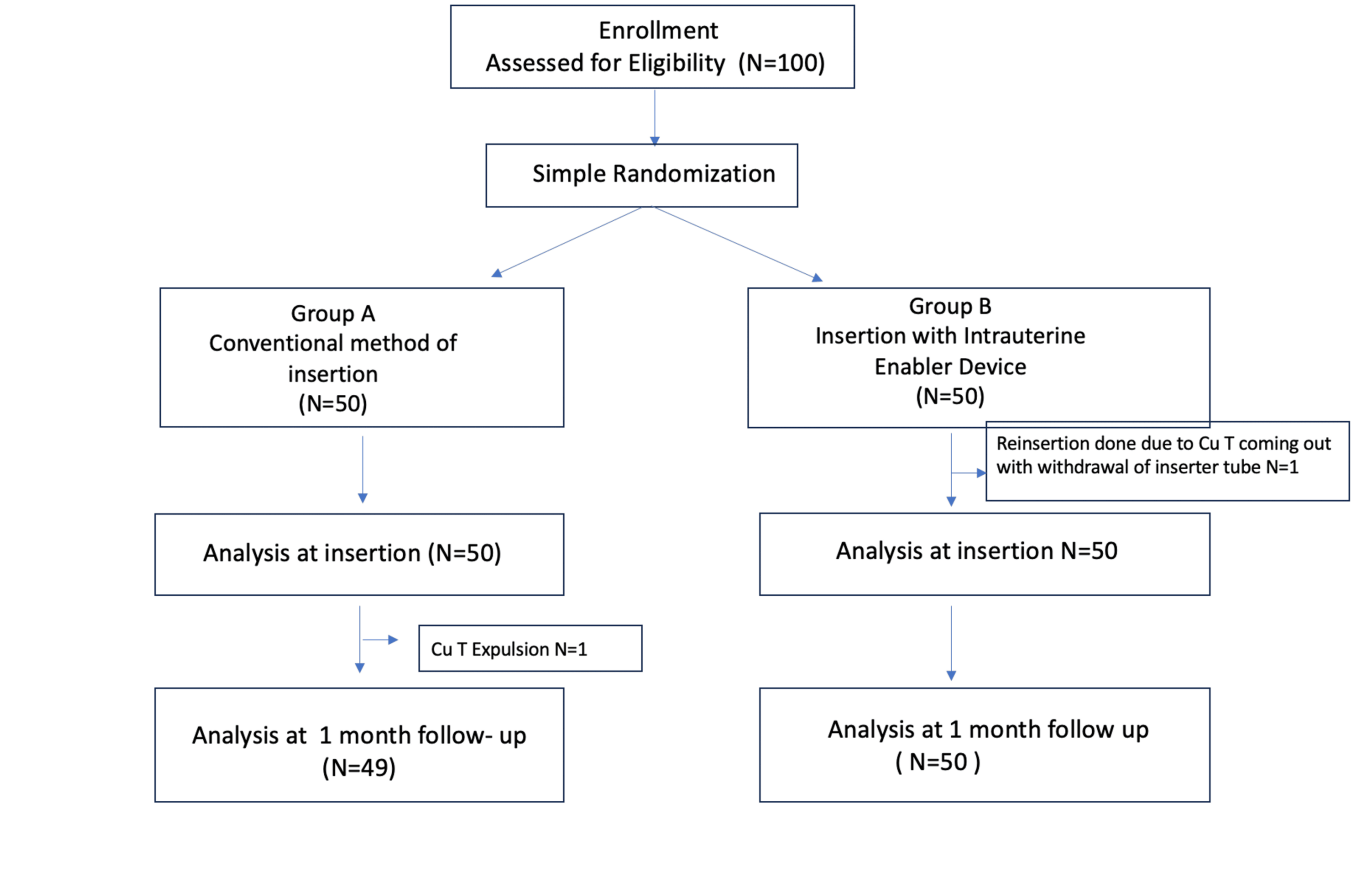
CONSORT flow diagram CONSORT, Consolidated Standards of Reporting Trials

Participants were followed up clinically and with ultrasound after their first period or at one-month post-insertion, at which point the distance from the superior end of the Cu-IUD to the inner uterine wall was recorded. The Cu-IUD and Etherena were provided free of charge by Pregna International Ltd., with no costs incurred by participants for the IUD, its insertion, or follow-up. Both the clients and the statistician were blinded to the insertion technique used in the two groups. Figure 3 shows the Consolidated Standards of Reporting Trials (CONSORT) flow diagram. The study protocol can be accessed by contacting the corresponding author.

Outcome measures included the provider’s ease of loading the arms of the Cu-IUD, the measurement of uterocervical length on the inserter tube, the technique of placement, insertion time, and overall handling of the device. Additionally, the study compared the distance from the superior end of the Cu-IUD to the endometrial verge immediately after the procedure and at one month of follow-up.

Statistical analysis

Demographic and clinical details, along with client and provider acceptability data, were recorded for each participant. Data entry was performed using Microsoft Excel (Microsoft Corporation, Redmond, WA, USA), and the final analysis was conducted with IBM SPSS Statistics for Windows, Version 25.0 (Released 2017; IBM Corp., Armonk, NY, USA). Data between the two groups were compared, with categorical variables presented as numbers and percentages (%). Quantitative data with non-normal distribution were reported as medians with 25th and 75th percentiles (IQR). The normality of the data was assessed using the Kolmogorov-Smirnov test. For non-normally distributed data, nonparametric tests were utilized. The Mann-Whitney test was employed for comparing quantitative variables between the two groups, while the Wilcoxon signed-rank test was used for comparisons across follow-up. Qualitative variables were analyzed using the chi-square test; if any cell had an expected value of less than 5, Fisher’s exact test was applied. A p-value of less than 0.05 was considered statistically significant.

## Results

A total of 100 women were evaluated for eligibility, as shown in the CONSORT flow diagram (Figure 3). Participants were randomly assigned to two groups: Group A, comprising 50 women who underwent Cu-IUD insertion using the conventional technique, and Group B, consisting of 50 women who received Cu-IUD insertion using the Intrauterine Enabler Device. There were no losses to follow up.

Baseline characteristics

A comparison of the baseline characteristics between the two groups was conducted, and both groups were found to be comparable (Table 1).

**Table 1:** Comparison of sociodemographic characteristics between Group A (conventional insertion) and Group B (intrauterine enabler device) ^†^ Chi-square test ^‡^ Mann-Whitney test

Sociodemographic characteristics	Group A – Conventional insertion (n = 50)	Group B – Intrauterine enabler device (n = 50)	p-value
Parity			
Primiparous	11 (22%)	9 (18%)	0.61^†^
Multiparous	39 (78%)	41 (82%)	
Age (years) (mean ± SD)	29.5 ± 3.9	31 ± 4.66	0.13^‡^

All insertions were performed by two faculty members and four residents, with the majority conducted by the residents. Specifically, only nine insertions in Group A and 11 insertions in Group B were carried out by the faculty.

Provider and participant experience

The comparison of provider and participant experiences between the two groups is presented in Table 2.

**Table 2:** Comparison of the provider and participant experience between Group A (conventional insertion) and Group B (intrauterine enabler device) ^*^ Fisher’s exact test ^‡^ Mann-Whitney test

Outcome	Group A – Conventional insertion (n = 50)	Group B – Intrauterine enabler device (n = 50)	p-value
Loading of T arms, n (%)			
Easy	13 (26%)	47 (94%)	<0.0001^*^
Difficult	37 (74%)	3 (6%)	
Setting measurement of uterocervical length on inserter tube, n (%)			
Easy	45 (90%)	50 (100%)	0.05^*^
Difficult	5 (10%)	0 (0%)	
Technique of placement, n (%)			
Easy	43 (86%)	50 (100%)	0.012^*^
Difficult	7 (14%)	0 (0%)	
Overall handling of the device, n (%)			
Easy	32 (64%)	49 (98%)	<0.0001^*^
Difficult	18 (36%)	1 (2%)	
Perforation/injury, n (%)			
No	50 (100%)	50 (100%)	NA
Yes	0 (0%)	0 (0%)	
Time of insertion (in seconds)	180 (180-210)	120 (120-120)	<0.0001^‡^
Participant comfort during sounding, n(%)			
Comfortable	22 (44%)	48 (96%)	<0.0001^*^
Painful	28 (56%)	2 (4%)	
Feedback of participants after insertion, n (%)			
Satisfied	47 (94%)	48 (96%)	1^*^
Unsatisfied	3 (6%)	2 (4%)	

In Group B, 94% of doctors found loading the arms of the Cu 380 mm² IUD easy, compared to only 26% in Group A (p < 0.0001). Setting the uterine depth measurement on the inserter tube was consistently easy in Group B (100%), whereas it was easy 90% of the time in Group A (p = 0.054). The technique for placing the Cu 380 mm² IUD was easy every time in Group B, compared to 86% of the time in Group A (p = 0.012), with this difference being statistically significant. Overall handling of the device was significantly easier with the intrauterine enabler (98%) compared to the conventional method (64%) (p ≤ 0.0001). The median insertion time for the Cu 380 mm² IUD, including cervix stabilization, loading, and insertion, was significantly shorter with the Intrauterine Enabler (120 seconds) compared to the conventional method (180 seconds) (p < 0.0001). No cases of injury or perforation occurred with either technique.

Only 2% of participants experienced pain with sounding using the Esa, compared to 28% with the metallic sound, indicating a statistically significant difference (p < 0.0001). Nearly all participants (94% in Group A and 96% in Group B) expressed satisfaction with the procedure, showing no significant difference in satisfaction (p = 1). Additionally, no participants reported infection-related complaints during follow-up.

Comparison of ultrasound findings

The comparison of the distance from the superior end of the Copper 380 mm² IUD to the endometrial verge between the two groups, both immediately after insertion and during the follow-up visit, is presented in Table 3.

**Table 3:** Comparison of USG findings in mm (distance of the superior end of Cu T from endometrial verge) between Group A (conventional insertion) and Group B (intrauterine enabler device) ‡ Mann-Whitney test * There was one expulsion in Group A after 20 days of insertion. USG, ultrasonography

USG findings in mm (distance of the superior end of Cu T from endometrial verge)	Group A – Conventional insertion	Group B – Intrauterine enabler device	p-value
Distance at insertion	2 (1-3) (n = 50)	2 (1-3) (n = 50)	0.55^‡^
Distance at follow-up	3 (1-3) (n = 49)^*^	2 (1-3) (n = 50)	0.84^‡^

The median distance of the Cu 380 mm² IUD from the endometrial verge was 2 mm (1-3) at insertion for both techniques. At the follow-up visit, the median distance was 3 mm (1-3) in Group A and remained 2 mm (1-3) in Group B, with no significant difference observed. Notably, one case of expulsion occurred in Group A after 20 days of insertion.

## Discussion

What is known?

The “no-touch technique” recommended by the WHO is a globally accepted standard for preventing infection during the insertion of the Cu 380 mm² IUD. A critical aspect of this technique involves loading the arms of the Cu 380 mm² IUD into the inserter tube while keeping it in its sterile packaging, ensuring that it does not come into contact with any non-sterile surfaces. However, this step is often considered one of the most cumbersome aspects of the Cu 380 mm² IUD insertion process.

Findings and interpretations

The intrauterine enabler device Etherena has been designed to simplify the insertion of the Cu 380 mm² IUD, making the procedure more acceptable for providers and less painful for clients. In our study, significantly more participants (96%) in Group B reported comfort during the sounding step compared to only 44% in Group A. The soft, smooth material and rounded tip of the Etherena sound facilitate easier sounding compared to the metallic sound. Moreover, a majority of providers (94%) found loading the Cu 380 mm² IUD with Etherena easy, whereas 74% of providers using the conventional method found this step challenging, with two requiring assistance from senior staff.

Setting the measurement on the uterine inserter tube was straightforward for all doctors (100%) using Etherena, compared to 90% using the conventional method. Insertion times were significantly reduced, with a median difference of 60 seconds favoring Etherena. The overall handling and placement technique with Etherena were reported as easy by 100% and 98% of providers, respectively, in contrast to 86% and 64% with the conventional method.

Consequently, we conclude that the insertion of the Cu 380 mm² IUD using Etherena is substantially easier for providers compared to the no-touch technique. Safety was maintained, with no instances of perforation, injury, or infection reported in either technique. Thus, the intrauterine enabler device significantly enhanced the provider experience, reduced insertion time, and maintained patient safety and satisfaction.

A similar study by Maheshwari et al. in 2021 [9] corroborated our findings, concluding that the intrauterine enabler device offers a safe and innovative approach, making Cu IUD insertion more convenient for providers while significantly enhancing client comfort. However, in our study, we compared the distance of the superior end of the Cu 380 mm² IUD from the endometrial verge rather than from the fundus, as myometrial thickness may vary among patients. Tangtono et al. defined an IUD as displaced when post-insertion transvaginal ultrasound indicated a distance of ≥3 mm from the superior edge of the IUD to the endometrial verge measured in a sagittal plane [10]. It is well established that a greater distance of the IUD from the uterine fundus increases the likelihood of complications, including pelvic pain, heavy bleeding, and higher expulsion rates [11,12].

In our study, the distance measured post-insertion with Etherena was comparable to that of the conventional method, averaging 2 mm (1-3) at both insertion and one-month follow-up (Table 3). This indicates that the novel method achieves effective fundal placement of the Cu 380 mm² IUD, similar to the conventional technique.

Overall, significantly more doctors expressed satisfaction, and the insertion of the Cu 380 mm² IUD was deemed easier with Etherena compared to conventional methods. Although fewer participants experienced pain with Etherena, satisfaction levels were similar across both methods, as participants prioritized the successful outcome of IUD insertion. However, a notable drawback of the Etherena is its cost, as the Cu 380 mm² IUD is typically available for free in Indian public health facilities. A comparable study evaluated the safety, feasibility, and acceptability of a novel inserter for postpartum IUDs and found that this long inserter was effective, convenient, comfortable, and safe for postpartum IUD insertion [13].

Strength and limitations

Our study is a double-blinded, randomized trial comparing a novel technique with a standard, widely accepted method. We recorded the experiences of both participants and providers throughout the procedure. However, one limitation of our study is the inability to assess overall pain experienced during the entire procedure using a Visual Analogue Scale, which would have provided an objective measure of the client’s experience. Additionally, we did not exclude patients with known psychiatric disorders, which may affect the results. Furthermore, this pilot investigation of a novel device highlights the need for larger trials to evaluate its performance across a broader range of patients and providers.

## Conclusions

The “no-touch technique,” endorsed by the WHO, is widely considered the gold standard for IUD insertion due to its focus on minimizing infection risk and ensuring proper aseptic conditions. However, the novel intrauterine enabler device, such as the Etherena T Cu 380, offers a viable alternative to this standard technique. This new device facilitates easier and more precise insertion of the Cu 380 mm² IUD. Based on observed outcomes, insertion with the Etherena T Cu 380 appears not only easier but also equally safe and effective compared to the conventional no-touch technique.

For healthcare providers, this device enhances the overall experience of IUD insertion by simplifying the procedure and reducing technical challenges. For clients, the use of this novel device is associated with reduced discomfort and pain during insertion. Thus, the Etherena T Cu 380 represents a promising advancement that streamlines the IUD insertion process, making it more efficient and patient-friendly while upholding the safety and efficacy standards set by the WHO.
